# A simple prognostic score to predict recurrence after pancreaticoduodenectomy for ampullary carcinoma: results from the French prospective FFCD-AC cohort

**DOI:** 10.1016/j.esmoop.2024.103988

**Published:** 2024-11-18

**Authors:** G. Roth, A. Pellat, G. Piessen, K. le Malicot, L. Schwarz, C. Gallois, D. Tougeron, V. Hautefeuille, M. Jary, S. Benoist, M. Amil, R. Desgrippes, M. Muller, T. Lecomte, M. Guillet, C. Locher, C. Genet, S. Manfredi, O. Bouché, J. Taieb

**Affiliations:** 1 University Grenoble Alpes/Hepato-Gastroenterology and Digestive Oncology Department, CHU Grenoble Alpes/Institute for Advanced Biosciences, CNRS UMR 5309-INSERM U1209, Grenoble, France; 2 Service de gastroentérologie, d’endoscopie et d’oncologie digestive, Hôpital Cochin, APHP, Paris, France; 3 Centre of Research in Epidemiology and Statistics (CRESS), Université Paris Cité, INSERM U1153, Paris, France; 4 University of Lille/Cancer Heterogeneity Plasticity and Resistance to Therapies, UMR9020-U1277 INSERM-CNRS/Digestive Surgery Department, CHU Lille, Lille, France; 5 Fédération Francophone de Cancérologie Digestive (FFCD), EPICAD INSERM LNC-UMR 1231, Faculté de Médecine, University of Burgundy and Franche Comté, Dijon, France; 6 Department of Digestive Surgery, Charles Nicolle Hospital, Rouen, France; 7 Institut du Cancer Paris CARPEM, APHP, Hepatogastroenterology and GI Oncology Department, APHP Centre-Université Paris Cité, Hôpital Européen G. Pompidou, Paris, France; 8 Hepato-Gastroenterology Department, Poitiers University Hospital, Poitiers, France; 9 Department of Gastroenterology and Digestive Oncology, CHU Amiens-Picardie, Amiens, France; 10 Department of Digestive Oncology and Hepatobiliary Surgery, CHU Estaing, Clermont-Ferrand, France; 11 Department of Digestive Surgery and Surgical Oncology, Bicêtre Hospital, AP-HP, Paris-Saclay University, Le Kremlin Bicêtre, France; 12 Department of Hepatology, Gastroenterology and Digestive Oncology, Centre Hospitalier de Vendée, La Roche sur Yon, France; 13 Department of Hepatology, Gastroenterology and Digestive Oncology, Saint Malo General Hospital, Saint Malo, France; 14 Department of Gastroenterology, Nancy University Hospital, University of Lorraine, Nancy, France; 15 Hepatogastroenterology Department, University Hospital, Tours, France and INSERM UMR 1069, Tours University, Tours, France; 16 Department of Gastroenterology and Digestive Oncology, Hospices civils de Lyon, CHU de la Croix Rousse, Lyon, France; 17 Gastroenterology and Digestive Oncology Department, Meaux General Hospital, Meaux, France; 18 Oncology Department, Louis Pasteur Hospital, Chartres, France; 19 Hepatology and Gastroenterology Unit, Dijon University Hospital/INSERM U1231/University of Burgundy Dijon, Dijon, France; 20 Gastroenterology and Digestive Oncology Department, Université Reims Champagne Ardenne, CHU Reims, Reims, France

**Keywords:** ampullary carcinoma, adjuvant therapy, tumor recurrence, prognostic score

## Abstract

**Background:**

Ampullary carcinoma (AC) is a rare and severe gastrointestinal cancer with a disease recurrence rate of around 40% after curative-intent surgery and for which the main prognostic factors and adjuvant treatment decision remain a matter of debate.

**Patients and methods:**

The FFCD-AC cohort is a French nationwide prospective cohort, which included patients with non-metastatic resected AC. The primary objective of this study was to describe prognostic factors associated with disease-free survival (DFS) and overall survival (OS) after pancreaticoduodenectomy (PD) so as to propose a user-friendly score to better estimate the risk of recurrence. The secondary objective was to study the benefit of adjuvant therapy in terms of DFS and OS.

**Results:**

Three hundred and seventy patients with resected AC were included. Median follow-up was 40.6 months. Median age was 68.5 years (32.0-87.0 years), 53.8% of patients were male and 56.1%/37.4%/6.5% had an Eastern Cooperative Oncology Group performance status 0/1/2, respectively. Pathological subtype was intestinal/pancreatobiliary/mixed-undetermined in 29.5%/40.5%/30.0% of patients, respectively. Adjuvant chemotherapy was carried out in 61% of patients. In multivariable analysis, stage III tumor [hazard ratio (HR) 2.86, (95% confidence interval {95% CI}: 1.89-4.17), *P* < 0.0001], high tumor grade [HR 2.51, (95% CI: 1.42-4.43), *P* = 0.002] and non-intestinal subtype [HR 1.58, (95% CI: 1.00-2.49), *P* = 0.052] were associated with shorter DFS. A score based on these three parameters divided patients into low (*n* = 83), intermediate (*n* = 133) and high risk (*n* = 96) with median DFS not reached (NR)/73.1/15.2 months and a median OS NR/86.1/38.2 months, respectively. After propensity score matching, adjuvant chemotherapy was associated with longer DFS [HR 0.57, (95% CI: 0.45-0.72), *P* < 0.0001] in the cohort.

**Conclusion:**

Our integrated score based on three easy-to-collect items—lymph node invasion, tumor grade and non-intestinal subtypes—seems highly prognostic in resected AC and needs to be confirmed in an external validation dataset to help adjuvant treatment decision making.

## Highlights

• AC is a rare disease with very heterogeneous practices worldwide due to the absence of a formal standard.
• FFCD-AC is a French nationwide prospective cohort of 370 patients resected by PD for an AC.
• Two-year DFS was 62%, aligned with previous cohorts and 61% of patients received adjuvant therapy.
• FFCD-AC proposes a user-friendly score to predict recurrence based on tumor subtype, tumor grade and tumor stage.
• After propensity score, FFCD-AC suggests that adjuvant therapy is associated with improved DFS and OS.

## Introduction

Ampullary carcinoma (AC) is a rare disease accounting for 0.2% of gastrointestinal (GI) cancers. Due to the anatomical location of the ampulla of Vater at the confluence of the duodenum, the main bile duct and the main pancreatic duct, AC corresponds to a heterogeneous group of cancers divided into three subtypes with different morphological patterns and prognostic profiles, as follows: intestinal (30%-40% of cases), pancreatobiliary (45%-60%) and mixed, also sometimes called undetermined (10%-20%).^1^, ^2^, ^3^, ^4^ As it often leads to early main bile duct obstruction with jaundice, and due to its distance from arterial axes such as the coeliac trunk or the superior mesenteric artery, AC is more often accessible to resection than other periampullary cancers such as pancreatic cancer. However, even in resected patients, prognosis is still poor with 2-year disease-free survival (DFS) rates ranging from 50% to 66.2%^5^^,^^6^ and 5-year overall survival (OS) rates ranging from 52% to 67.9%,^1^^,^^2^^,^^5^, ^6^, ^7^ without significant improvement observed in the last 30 years.^8^

The place of adjuvant therapy after curative-intent resection is still debated as no standard of care has been fully established due to the limited number of patients experiencing AC. To date, ESPAC3 is the only phase III trial which attempted to prove the benefit of single-agent adjuvant therapy in 428 patients with resected periampullary tumors including 297 AC.^6^ No significant benefit for OS or DFS was seen, though a clinically relevant trend was observed. However, due to the lack of a robust prospective trial, the use of adjuvant therapy in AC remains debated.^6^ Retrospective cohort studies have suggested that the benefit of adjuvant therapy might vary according to various factors. Among these, tumor stages T and N, tumor grade, AC subtypes and invasion of resection margins have been suggested. However, the most relevant factors for determining the risk of disease recurrence and guiding clinicians in their decision to prescribe adjuvant treatment remain to be defined. Several scores have been constructed from retrospective cohorts such as the one developed by Moekotte et al. who proposed a prediction model based on age, resection margin, tumor grade and TNM (tumor–node–metastasis) staging.^2^ In a retrospective cohort of 152 resected patients, Colussi et al. developed a score integrating TNM stage, age, World Health Organization performance status and tumor grade dividing patients into three prognostic groups with a 5-year DFS of 73.5% in the low-risk group dropping to 20.1% in the high-risk group.^5^ In the high-risk group, the 5-year DFS was 29.2% among patients who received adjuvant chemotherapy and 8.3% among those who did not (*P* = 0.32). However, as 10-15 years ago the pathological subtype of the tumor was not mentioned in all pathological reports, none of these scores integrated this potential important prognosticator.^1^^,^^6^

In this article, we propose an integrated score based only on basic post-operative pathological parameters such as TNM stage, tumor grade and pathological subtype to easily estimate the risk of recurrence and to help decision making regarding adjuvant treatment.

## Patients and methods

### Study design and patient selection

The FFCD-AC cohort is a prospective French cohort of patients surgically resected for an AC. Patients were eligible if they were aged 18 and over, and have been resected for a non-metastatic AC without macroscopic residual tumor residue (R2) within 1 year before inclusion. Non-inclusion criteria were: non-ampullary tumors, ampullary tumors other than adenocarcinoma and metastatic or unresectable locally advanced AC at diagnosis. In this study, only patients resected by pancreaticoduodenectomy (PD) were eligible.

### Study objectives

The primary objective of this study was to describe prognostic factors associated with DFS after PD so as to propose a user-friendly score to better estimate the risk of disease recurrence. Secondary objectives were the relation between these prognostic factors and OS, and to evaluate the impact of adjuvant therapy on survival outcomes.

### Data collection

Baseline patient characteristics including general condition, comorbidities, tumor and treatment features were prospectively recorded in the FFCD e-CRF (ramdam.ffcd.fr). pTNM stage based on the American Joint Committee on Cancer (AJCC) eighth edition,^9^ pathological subtype and tumor grade (low-, intermediate- and high-grade tumors corresponding to well-differentiated, moderately differentiated and poorly differentiated/undifferentiated tumors, respectively) were locally assessed and data were collected from surgical and pathological reports. Regarding pathological subtype, immunohistochemistry (IHC) was carried out according to local practice and patients were categorized as intestinal, pancreatobiliary or mixed/undetermined subtypes. Follow-up data including disease progression events, death events and subsequent treatments in the case of relapse were prospectively collected until patient’s death or the end of the study.

### Ethics

This study was validated by the independent ethics committee CPP Ile-de-France VIII and covered by a declaration to the ‘Commission nationale de l'informatique et des libertés’ (CNIL). Patients were orally informed and were given a document summarizing the objectives, modalities and confidentiality rules of the study with a non-opposition form. The cohort was registered on ClinicalTrials.gov (NCT03800212).

### Statistical analysis

Quantitative variables were described by the usual descriptive statistics: mean, standard deviation, median, interquartile range, minimum and maximum. The qualitative variables were described using number and percentages. Comparisons between groups were carried out by means of Student’s *t*-test or the Wilcoxon test (according to the distribution of the variables) for quantitative variables and the chi-square test or Fisher’s exact test for qualitative variables. Confidence intervals were 95% (95% CI) two-sided intervals. OS and DFS were plotted using the Kaplan–Meier estimator. Survival rates at different times were calculated and their 95% CIs were also estimated. The assumption of risk proportionality and log-linearity was verified for each variable. Univariate and multivariate analyses were done using the Cox model to determine prognostic factors of OS and DFS. Multivariate analyses included variables with *P* < 0.10 in univariate analyses.

The propensity score was derived from an unconditional multivariate logistic regression which estimates the probability of receiving adjuvant therapy using variables with a *P* value < 0.05 in univariate logistic regression. Performance and adequacy of the model were checked with the area under the curve (AUC) and the Hosmer–Lemeshow test, respectively. We then applied in a univariate Cox model the inverse of probability of treatment weighting (IPTW) method using the propensity score, hereinafter called ‘weighted sample’.

The prognostic score for a given patient was a combination of the different variables included in the multivariate analysis, weighted with their regression coefficients. The score was calibrated so that each variable contributed 0, 1, 2 or 3 units. The score was then calculated for each patient by adding the points corresponding to each prognostic factor. The predictive value and discriminatory capacity of the score was validated by calculating Harrel’s C discrimination index extended to survival data. Harrell’s C index ranges from 0.5 (no discrimination) to 1.0 (perfect discrimination). Statistical analyses were conducted using SAS software version 9.4.

## Results

### Patient characteristics

Three hundred and eighty-nine patients who underwent resection of an AC were prospectively included in the FFCD-AC cohort between July 2014 and June 2023 by 50 French centers. Among them, 370 were resected by PD and eligible for this study (19 were resected by papillectomy only and excluded). In these 370 patients, median age was 68.5 years (min-max: 32.0-87.0 years) and 199 (53.8%) were male. The Eastern Cooperative Oncology Group (ECOG) performance status was 0, 1 or 2 in 180 (56.1%), 120 (37.4%) and 21 (6.5%) patients, respectively.

TNM stage was 0, I, II or III in 7 (1.9%), 100 (27.1%), 65 (17.6%) and 197 (53.4%) patients, respectively, with R0 resection margins in 359 (97.3%) patients. Pathological subtype based on morphological examination and IHC analyses was intestinal, pancreatobiliary and mixed/undetermined in 109 (29.5%), 150 (40.5%) and 111 (29.0%) patients, respectively. Adjuvant chemotherapy was carried out in 226 (61.1%) patients. Detailed patient characteristics are given in Table 1 and Supplementary Table S1, available at https://doi.org/10.1016/j.esmoop.2024.103988.

**Table 1:** Patient characteristics

Patient characteristics	Overall cohort (*n* = 370)
Age, median (min-max)	68.5 (32.0-87.0)
Sex, *n* (%)	
Male	199 (53.8%)
Female	171 (46.2%)
ECOG performance status, *n* = 321, *n* (%)	
0	180 (56.1%)
1	120 (37.4%)
2	21 (6.5%)
Body mass index (kg/m^2^), *n* = 367, median (IQR)	29.5 (26.2-33.7)
pTNM Stage, *n* (%)	
0	7 (1.9%)
I	100 (27.1%)
II	65 (17.6%)
III	197 (53.4%)
Resection margin, *n* = 369, *n* (%)	
R0	359 (97.3%)
R1	10 (2.7%)
Pathological subtype, *n* = 370, *n* (%)	
Intestinal	109 (29.5%)
Pancreatobiliary	150 (40.5%)
Mixed/Undetermined	32 (8.6%)/79 (21.4%)
Not determined	79 (21.4%)
Tumor grade, *n* = 370, *n* (%)	
Low	103 (27.8%)
Intermediate	166 (44.9%)
High	44 (11.9%)
Undetermined	57 (15.4%)
MMR status, *n* = 134, *n* (%)	
MSI-high/dMMR	13 (9.7%)
MSS/pMMR	121 (90.3%)
Adjuvant chemotherapy, *n* = 370, *n* (%)	
No	144 (38.9%)
Yes	226 (61.1%)
Single-agent	73 (32.3%)
Doublet/triplet	153 (67.7%)

dMMR, deficient mismatch repair; ECOG, Eastern Cooperative Oncology Group; IQR, interquartile range; MSI, microsatellite instability; MSS, microsatellite stability; pMMR, proficient MMR; TNM, tumor–node–metastasis.

### Survival outcomes

At the time of the analysis, the median follow-up of the cohort was 40.6 months (95% CI: 34.7-45.2 months). Median DFS (mDFS; *n* = 151 events) was 47.3 months (35.1-87.7 months) with 12-, 24- and 48-month DFS rates of 78.8% (95% CI: 74.0%-82.8%), 62.1% (56.3-67.3) and 49.7% (43.2-55.8), respectively. First disease recurrence was locoregional in 19 (15.7%) patients, and metastatic in 102 (84.3%) patients. Median OS (mOS; *n* = 114 events) was 81.5 months [63.3 months-not reached (NR)] with 12-, 24- and 48-month OS rates of 92.9% (89.7%-95.2%), 81.0% (76.0%-85.1%) and 60.7% (54.0%-66.7%), respectively.

### Uni- and multivariable analysis

In univariable analysis, factors associated with significantly poorer DFS were: age ≥75 [hazards ratio (HR) 1.42 (1-2.02), *P* = 0.048], ECOG performance status ≥1 [HR 1.56 (1.1-2.22), *P* = 0.013], stage III disease [HR 2.63 (1.9-3.8), *P* < 0.0001], high tumor grade [HR 2.50 (1.46-4.29), *P* = 0.001]), mixed/undetermined [HR 2.05 (1.29-3.23), *P* = 0.004] and pancreatobiliary [HR 2.21 (1.43-3.43), *P* < 0.001] pathological subtypes (Table 2). In multivariable analysis, only high-grade tumor [HR 2.51 (1.42-4.43), *P* = 0.002], stage III disease [HR 2.86 (1.89-4.17), *P* < 0.0001] and non-intestinal subtype [HR 1.58 (1.00-2.49), *P* = 0.052] were associated with shorter DFS (Table 2).

**Table 2:** Relationship between prognostic factors, disease-free survival and overall survival after univariable and multivariable analyses

	Disease-free survival				Overall survival			
	Univariable analysis		Multivariable analysis		Univariable analysis		Multivariable analysis	
	HR (95% CI)	*P*	HR (95% CI)	*P*	HR (95% CI)	*P*	HR (95% CI)	*P*
Age (years)								
<75	1.00 (Ref)		1.00 (Ref)		1.00 (Ref)		1.00 (Ref)	
≥75	1.42 (1-2.02)	0.048	1.20 (0.79-1.82)	0.395	1.69 (1.14-2.51)	0.009	1.66 (1.03-2.66)	0.036
ECOG PS								
0	1.00 (Ref)		1.00 (Ref)		1.00 (Ref)		1.00 (Ref)	
≥1	1.56 (1.1-2.22)	0.013	1.45 (0.98-2.14)	0.066	1.48 (0.98-2.23)	0.059	1.20 (0.76-1.91)	0.438
TNM stage								
0-I-II	1.00 (Ref)		1.00 (Ref)		1.00 (Ref)		1.00 (Ref)	
III	2.63 (1.9-3.8)	<0.0001	2.86 (1.89-4.17)	<0.0001	2.44 (1.6-3.7)	<0.0001	2.63 (1.67-4.17)	<0.0001
Tumor grade								
Low	1.00 (Ref)		1.00 (Ref)		1.00 (Ref)		1.00 (Ref)	
Intermediate	1.49 (0.97-2.3)	0.067	1.24 (0.78-1.99)	0.368	1.55 (0.94-2.56)	0.084	1.41 (0.81-2.45)	0.224
High	2.50 (1.46-4.29)	0.001	2.51 (1.42-4.43)	0.002	2.79 (1.53-5.09)	0.001	2.81 (1.48-5.32)	0.002
Undetermined	2.11 (1.27-3.52)	0.004	1.95 (1.09-3.5)	0.025	1.73 (0.92-3.23)	0.087	1.62 (0.77-3.37)	0.202
Pathological subtype								
Intestinal	1.00 (Ref)		1.00 (Ref)		1.00 (Ref)		1.00 (Ref)	
Non-intestinal	2.14 (1.42-3.22)	<0.001	1.58 (1-2.49)	0.052	1.99 (1.24-3.19)	0.005	1.38 (0.81-2.33)	0.234
Adjuvant therapy								
No adjuvant	1.00 (Ref)				1.00 (Ref)			
Adjuvant (all types)	1.09 (0.78-1.53)	0.600			1.04 (0.71-1.52)	0.835		
Single-agent	1.35 (0.9-2.04)	0.149			1.25 (0.79-1.98)	0.342		
Doublet/triplet	0.96 (0.66-1.4)	0.848			0.92 (0.59-1.41)	0.690		

CI, confidence interval; ECOG, Eastern Cooperative Oncology Group; HR, hazard ratio; PS, performance status; Ref, reference; TNM, tumor–node–metastasis.

Regarding OS, in univariable analysis, similar factors were associated with poorer OS: age ≥75 [HR 1.69 (1.14-2.51), *P* = 0.009], ECOG ≥1 [HR 1.48 (0.98-2.23), *P* = 0.059], stage III disease [HR 2.44 (1.6-3.7), *P* < 0.0001], high tumor grade [HR 2.79 (1.53-5.09), *P* = 0.001], mixed/undetermined [HR 1.86 (1.09-3.15), *P* = 0.02] and pancreatobiliary [HR 2.10 (1.27-3.49), *P* < 0.004] pathological subtypes (Table 2). In multivariable analysis, only age ≥75 [HR 1.66 (1.03-2.66), *P* = 0.04], stage III [HR 2.63 (1.67-4.17), *P* < 0.0001] and high tumor grade [HR 2.81 (1.48-5.32), *P* = 0.002] were independently associated with shorter mOS (Table 2). DFS and OS curves according to TNM stage, pathological subtype and tumor grade are presented in Supplementary Figure S1, available at https://doi.org/10.1016/j.esmoop.2024.103988.

### Score to predict relapse and survival

**Figure 1:**
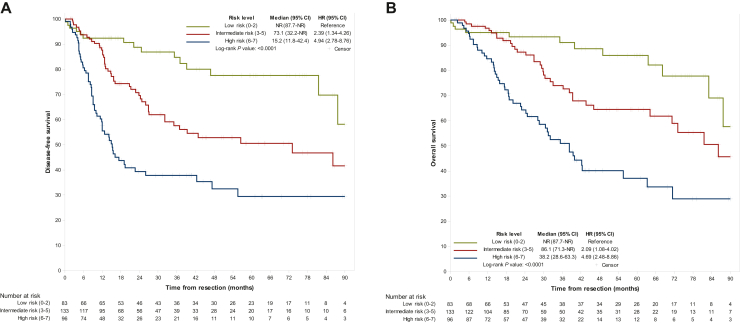
Survival outcomes according to the recurrence risk level. (A) Disease-free survival and (B) overall survival in the different risk level categories of the prognostication score. CI, confidence interval; HR, hazard ratio; NR, not reached.

Based on independent factors associated with shorter mDFS in multivariable analysis (tumor stage, tumor grade and tumor pathological subtype), a score to predict recurrence risk was constructed (Supplementary Table S2, available at https://doi.org/10.1016/j.esmoop.2024.103988). It allowed the distribution of patients into low- (0-2 points, *n* = 83 patients), intermediate- (3-5 points, *n* = 133 patients) and high-risk (≥6 points, *n* = 96 patients) groups with mDFS of NR (81.60 months-NR), 73.1 months (32.1 months-NR) and 15.2 months (11.3-22.6 months), respectively. Intermediate- and high-risk scores were associated with significantly shorter mDFS versus low-risk patients [HR_intermediate risk_ 2.39 (1.34-4.26), *P* = 0.003 and HR_high risk_ 4.94 (2.78-8.76), *P* < 0.0001] (Figure 1A, Table 3).

**Table 3:** Disease-free survival and overall survival according to different risk-level groups

	Disease-free survival			Overall survival		
**Risk level**	Low (*n* = 83)	Intermediate (*n* = 133)	High (*n* = 96)	Low (*n* = 83)	Intermediate (*n* = 133)	High (*n* = 96)
Median (95% CI) (months)	NR (81.6-NR)	73.1 (32.1-NR)	15.2 (11.3-22.6)	NR (83.1-NR)	86.1 (64.3-NR)	38.2 (28.2-55.6)
HR (95% CI)	Ref	2.39 (1.34-4.26)	4.94 (2.78-8.76)	Ref	2.09 (1.08-4.02)	4.69 (2.48-8.86)
*P*		0.003	<0.0001		0.03	<0.0001
Post-operative strategy						
Surveillance						
*N*	55	37	21	55	37	21
Median (95% CI) (months)	NR (87.69-NR)	39.26 (13.86-NR)	6.34 (4.47-9.92)	NR (87.69-NR)	64.26 (26.94-NR)	20.07 (7.39-39.95)
Adjuvant chemotherapy						
*N*	28	96	75	28	96	75
Median (95% CI) (months)	81.58 (46.78-NR)	86.14 (32.13-NR)	19.12 (14.06-47.34)	83.12 (63.61-NR)	86.14 (71.26-NA)	39.49 (28.16-71.43)

Median DFS and OS with HR and *P* value of high- and intermediate-risk patients are compared with low-risk patients considered as the reference. Overall outcomes of patients treated with adjuvant chemotherapy or surveillance only are presented for each level of risk.

CI, confidence interval; DFS, disease-free survival; HR, hazard ratio; NR, not reached; OS, overall survival; Ref, reference.

mOS in low-, intermediate- and high-risk patients was NR (83.1 months-NR), 86.1 months (64.3 months-NR) and 38.2 months (28.2-55.6 months), respectively. Intermediate- and high-risk scores were associated with significantly shorter mOS versus low-risk patients [HR_intermediate risk_ 2.09 (1.08-4.02), *P* = 0.03 and HR_high risk_ 4.69 (2.48-8.86), *P* < 0.0001] (Figure 1B, Table 3).

### Role of adjuvant therapy in AC

Among patients who received adjuvant chemotherapy (*n* = 226), 59 (26.1%) were treated with FOLFIRINOX, 61 (27.0%) with FOLFOX, 46 (20.4%) with gemcitabine, 26 (11.5%) with 5-fluorouracil, 16 (7.1%) with GEMCAP, 9 (4%) with GEMOX and 9 (4%) with another regimen. In univariable analysis, administration of adjuvant therapy was not associated with better DFS [HR 1.04 (0.71-1.52), *P* = 0.835] or OS [HR 1.09 (0.78-1.53), *P* = 0.60].

A propensity score, derived from multivariable logistic regression analysis, was determined to estimate the probability of receiving adjuvant therapy, including age, ECOG performance status, pTNM stage, tumor grade and pathological subtype. The AUC for a multivariate logistic model was equal to 0.7896 and the *P* value of the Hosmer–Lemeshow test was equal to 0.52. Results from multivariate Cox models for IPTW-weighted analysis showed statistically significant favorable survivals in patients receiving adjuvant chemotherapy compared to others with HR for DFS of 0.57 (95% CI 0.45-0.72, *P* < 0.0001) and for OS of 0.57 (95% CI 0.44-0.74, *P* < 0.0001).

Finally, even though statistical comparisons were not carried out, because of the low number of patients, we explored survival outcomes in different risk-level groups according to post-operative strategy. mDFS in patients in the low-, intermediate- and high-risk groups treated with adjuvant therapy were 81.58 months (46.78 months-NR), 86.14 months (32.13 months-NR) and 19.12 months (14.06-47.34 months), respectively, while mDFS for patients treated with surgery alone were NR (87.69 months-NR], 39.26 months (13.86 months-NR) and 6.34 months (4.47-9.92 months), respectively. mOS of low-, intermediate- and high-risk patients treated with adjuvant therapy were 83.12 months (63.61 months-NR), 86.14 months [71.26 months-NA) and 39.49 months (28.16-71.43 months) when patients treated with surgery alone showed mOS of NR (87.69 months-NR), 64.26 months (26.94 months-NR) and 20.07 months (7.39-39.95 months), respectively (Table 3).

## Discussion

The FFCD-AC cohort is, to the best of our knowledge, the largest prospective cohort dedicated to AC to date, with 370 patients resected by PD. With a median DFS of 47.3 months and a median OS of 81.5 months, survival outcomes were consistent with other previously published nationwide or international cohorts.^2^^,^^5^^,^^10^ In multivariable analyses, high tumor grade, lymph node involvement and non-intestinal tumor subtype were associated with significantly shorter DFS. Based on these three routinely collected markers, we constructed a prognostic score to assess recurrence risk and guide adjuvant therapy decision making. Patients were divided into three prognostic groups with significantly shorter DFS in intermediate- and high-risk versus low-risk patients with HR of 2.39 (1.34-4.26) and 4.94 (2.78-8.76), respectively. Similar results were observed for OS. As previously described,^1^, ^2^, ^3^^,^^5^^,^^7^ lymph node-positive status was the prognostic marker associated with the highest risk of disease recurrence in multivariable analysis and its presence systematically classified patients in at least the intermediate-risk class. Resection margins status has been described as a significant prognosticator in AC and integrated in the score of Moekotte et al.^2^ However, 20% of patients were R1 in their cohort while positive resection margins were found in <10% of patients in several other cohorts. With 2.5% of R1 patients, the prognostic value of this marker could not be studied in our study.

Intestinal subtype was clearly a good prognostic factor, while pancreatobiliary and mixed/undetermined subtypes were both associated with poor survival outcomes. However, this third subtype remains controversial regarding its prognostic value, which varies between an intermediate and a poor prognosis similar to pancreatobiliary subtype, as well as its frequency, which is usually around 20%-40%,^4^^,^^5^^,^^11^^,^^12^ but drops to 10% of tumors in some cohorts.^1^^,^^3^ According to recent guidelines, in the case of unclear subtype after morphological analyses, IHC should be systematically carried out to distinguish pancreatobiliary from intestinal subtypes,^11^ but it is still not representative of daily practice in many centers. Our score is the first to integrate pathological subtype, with distribution and prognostic profiles of subtypes consistent with the literature,^3^^,^^4^^,^^11^^,^^12^ making it reproducible in clinics.

One of the main goals of post-operative prognostication is to guide the decision regarding adjuvant therapy. In our cohort, 226 (61.1%) patients received an adjuvant treatment, which was probably guided by tumor-related characteristics such as pTNM stage, tumor grade, pathological subtype or resection margin status, but also by the patient’s medical condition, including age, nutritional and general status and post-operative complications. While previous scores included age or performance status, these factors were not associated with survival outcomes in our multivariable analyses, and were consequently not included in the prognostication score.^2^^,^^5^

When we explored survival outcomes according to post-operative strategy, adjuvant therapy was not associated with better survival outcomes in univariable analysis and was therefore not included in our multivariable analyses. These results may be due to multiple biases inherent to observational cohorts without standardization of therapeutic strategies and reflect the real-world setting of our study. Firstly, as in real life, chemotherapy regimens were heterogeneous and often guided by the pathological subtype. Gemcitabine and FOLFIRINOX were the most frequently used regimens for pancreatobiliary subtypes, and FOLFOX for intestinal subtypes (Supplementary Table S1, available at https://doi.org/10.1016/j.esmoop.2024.103988). Besides, no difference was observed between single-agent versus multiple-agent therapies in univariate analysis, even though few retrospective data support the idea that intensification of chemotherapy might prevent disease recurrence more effectively.^7^ This question is probably difficult to address in observational cohorts as adjuvant therapy decision making, as mentioned earlier, is influenced by many parameters, which compromises the comparability between patients. To overcome these biases, we carried out an IPTW propensity score showing that adjuvant therapy was independently associated with increased DFS [HR 0.57 (0.45-0.72), *P* < 0.0001] and OS [HR 0.57 (0.44-0.74), *P* < 0.0001]. This is concordant with previous findings, including those from the ESPAC3 trial, which is the only randomized phase III trial available to date.^2^^,^^6^^,^^7^ However, due to a limited number of patients without adjuvant therapy in intermediate- and high-risk groups, statistical comparisons were not carried out, but mDFS was 86.14 and 19.12 months in the case of adjuvant therapy and dropped to 39.26 and 6.34 months (Table 3) when surgery was the only treatment. Similar results were observed for OS, suggesting that adjuvant therapy could be beneficial in these patients. We now need randomized data to move forward in the adjuvant treatment of AC. The European phase III trial FFCD 2105/PRODIGE 98—AMPIRINOX is expected to start by the end of 2024 and will randomize mFOLFIRINOX versus single-agent chemotherapy (gemcitabine or capecitabine) in resected AC with stratification of pTNM stage, tumor subtype and tumor grade.

The strengths of our study are its multicentric and prospective design with a relatively high number of patients compared to previous publications on AC and a real-world setting showing the diversity of treatment options used in GI oncology departments all around the country. It allows to propose a pragmatic and simple score to prognosticate AC recurrence with classical pathological features which are now described in all standard pathology reports.

However, this study also presents several limitations mainly due to its observational character, with an important heterogeneity in disease and patient profiles, as well as therapeutic schedules and possible underreporting of some baseline variables. In addition, though this is one of the largest cohorts published on AC, the number of patients does not allow to have a sufficient statistical power for some subgroup analyses.

### Conclusion

AC is a rare disease with very heterogeneous practices worldwide due to the absence of formal standard adjuvant treatment regimen and indications. This study proposes a user-friendly score based on tumor subtype, tumor grade and TNM stage, which divides patients into low-, intermediate- and high-risk levels, linearly correlated with significant decreases in DFS and OS. Besides, after propensity score matching, this study suggests that adjuvant therapy is associated with improved survival outcomes. Though an external validation dataset would help to confirm these three parameters, our results suggest that these three important parameters should be stratified in future adjuvant trials, as has been agreed for the FFCD 2105/PRODIGE 98—AMPIRINOX ongoing trial.
