# Supplementary material for: A simple prognostic score to predict recurrence after pancreaticoduodenectomy for ampullary carcinoma: results from the French prospective FFCD-AC cohort
Source: ESMO Open. 2024 Nov 18;9(12):103988. doi: 10.1016/j.esmoop.2024.103988 (PMC11617226; doi:10.1016/j.esmoop.2024.103988)

Supplementary Figure 1: Survival outcomes according to pTNM stage, pathological subtype and tumor grade with DFS (A, B, C) and OS (D, E, F) in univariable analyses.


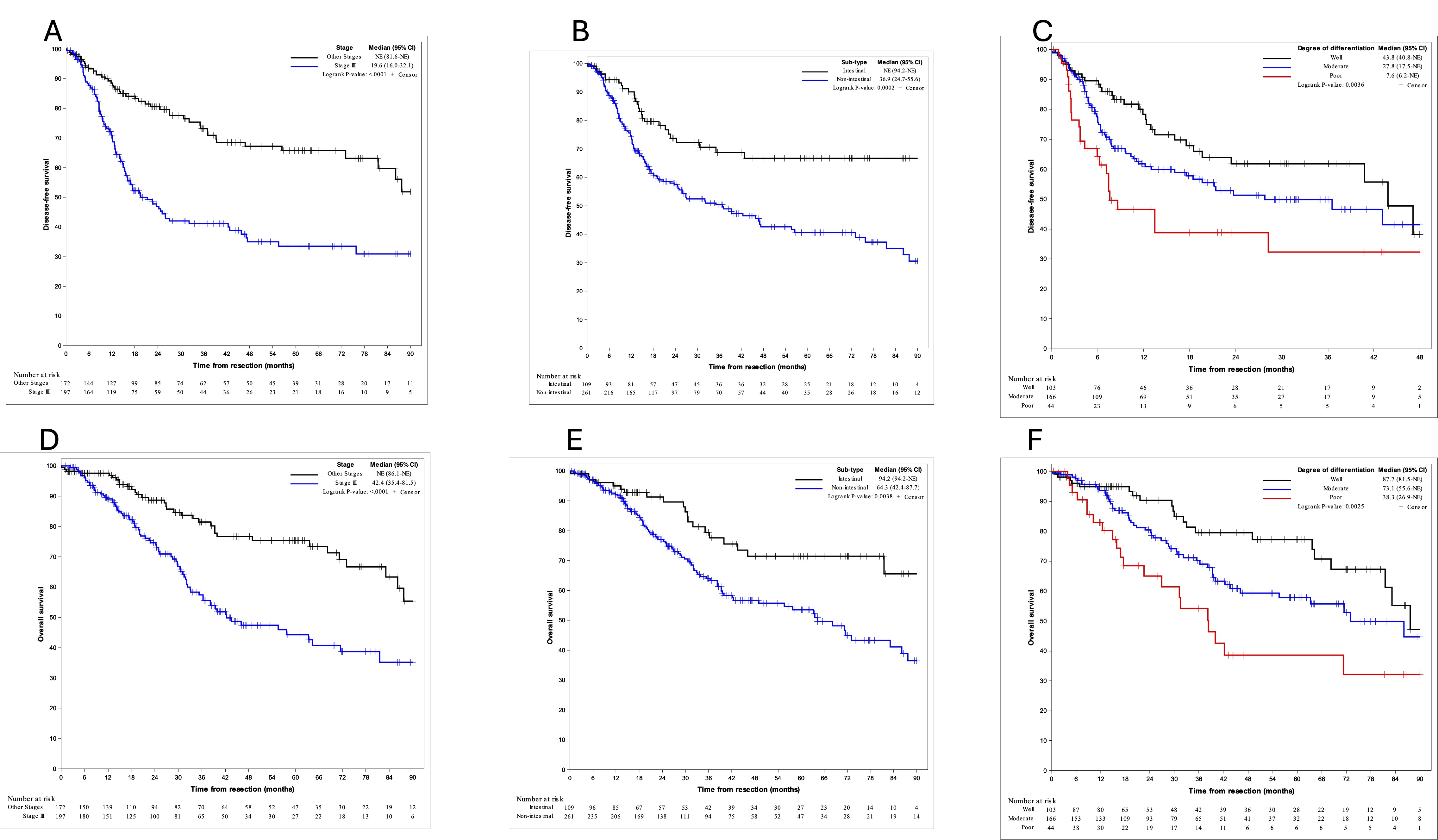

Supplement: Supplementary Fig [file mmc1.docx]
