# Supplementary material for: A simple prognostic score to predict recurrence after pancreaticoduodenectomy for ampullary carcinoma: results from the French prospective FFCD-AC cohort
Source: ESMO Open. 2024 Nov 18;9(12):103988. doi: 10.1016/j.esmoop.2024.103988 (PMC11617226; doi:10.1016/j.esmoop.2024.103988)
Supplement: Supplementary Tables [file mmc2.docx]

**Supplementary Table 1: Chemotherapy regimen distribution according to tumor subtypes.**

| Subtype | | Intestinal | Pancreato-biliary | Mixed/ undetermined | Total |
| --- | --- | --- | --- | --- | --- |
|  |  | (N=61) | (N=101) | (N=64) | (N=226) |
| Protocol | Gemcitabine | 7 (11.5%) | 23 (22.8%) | 16 (25.0%) | 46 (20.4%) |
|  | GEMOX | 3 (4.9%) | 5 (5.0%) | 1 (1.6%) | 9 (4.0%) |
|  | GEMCAP | 2 (3.3%) | 13 (12.9%) | 1 (1.6%) | 16 (7.1%) |
|  | LV5FU2 or capecitabine | 6 (9.8%) | 11 (10.9%) | 9 (14.0%) | 26 (11.5%) |
|  | FOLFOX | 38 (62.3%) | 7 (6.9%) | 16 (25.0%) | 61 (27.0%) |
|  | FOLFIRINOX | 3 (4.9%) | 37 (36.6%) | 19 (29.7%) | 59 (26.1%) |
|  | Other | 2 (3.3%) | 5 (5.0%) | 2 (3.1%) | 9 (4.0%) |

**Supplementary Table 2: Predictive score of recurrence**

| Variables | | Coefficient of variable in multivariable analysis mDFS (n=312) | Score weighting |
| --- | --- | --- | --- |
| Tumor stage | Stage I-II  Stage III | Ref  2.507 | 0  3 |
| Tumor grade | Low  Intermediate  High | Ref  1.275  2.281 | 0  1  2 |
| Tumor subtype | Intestinal  Non-intestinal | Ref  2.057 | 0  2 |
